# Parental Perception, Prevalence and Primary Care Physicians’ Knowledge on Childhood Food Allergy in Croatia

**DOI:** 10.3390/children2030305

**Published:** 2015-07-17

**Authors:** Tamara Voskresensky Baricic, Marija Catipovic, Erina L. Cetinic, Vlado Krmek, Ivona Horvat

**Affiliations:** 1 General Pediatric Office, Dom Zdravlja Zagreb-Centar, Runjaninova 4, 10000 Zagreb, Croatia; E-Mail: el.cetinic@gmail.com; 2 General Pediatric Office, Borisa Papandopula 26, 43000 Bjelovar, Croatia; E-Mail: mcatipov@inet.hr; 3 General Pediatric Office, Ante Starcevica 12, 12350 Metkovic, Croatia; E-Mail: vlado.krmek@du.t-com.hr; 4 University of Zagreb School of Medicine, Salata 4, 10000 Zagreb, Croatia; E-Mail: ivona.horvat@hotmail.com

**Keywords:** food allergy, children, parental perception, education on food allergy

## Abstract

Food allergy in children is increasing and the perception of food allergy among parents is even more common. In a questionnaire-based study of 702 children aged 6 to 48 months in four primary care settings, the aim was to determine the prevalence of perception *vs.* proven food allergy, parental anxiety and general pediatrician knowledge of food allergy. In 95/702 children (13.5%) parentally-reported food was associated reactions. IgE and/or skin prick test (SPT) and/or an open provocation test were performed in 48 (6.8%) and allergy was proven in 38 (5.4%) children. Discrepancy between parental perception and proven allergy is significant (*p* < 0.001), especially for food other than milk, egg and peanut (*p* < 0.001). Allergy to milk was the most common. Allergy to peanut was significantly more common in children ≥2 years (*p* < 0.05). Severe reactions occurred in 5/95 (5.2%) of all children and in 5/38 (13.1%) of allergic children, in 3/5 caused by peanut. Parents of children with proven allergy do not experience high degree of anxiety. The perception of food allergy among general pediatricians is limited, and in children with severe reactions precautionary measures and information to parents were insufficient. Parents and general pediatricians need additional education in food allergy.

## 1. Introduction

Food allergy is increasing in the developed world [1,2,3]. Most reactions in children occur to cow’s milk, egg, wheat, soy, tree nuts and fish, but differences in the prevalence of these specific foods are observed between Europe and other parts of the world [2,3,4,5]. The highest prevalence of food allergy in Europe is in Northwestern Europe [3,4,5]. It is highest in infants and toddlers; 2.5% of infants suffer from milk allergy, and up to 10% of 1-year-olds suffer from allergies to food, including cow’s milk, egg, nuts, soy, wheat, and fish [6,7,8]. Although population-based studies have been carried out in several European countries [6,9], this is the first report on food allergy perception and prevalence in Croatia. Self-reported adverse food reactions overestimate the prevalence of true food reactions in adults [10,11] and the same is reported for children [7,11,12]. The parental perception of child’s allergy depends on the child’s age, severity of reaction and education [13]. Proven food allergy is not an independent factor of parental distress and anxiety that reflects the quality of life [14,15]. Knowledge and education of primary care physicians on food allergy need to be improved, as there are, according to some studies [16], significant knowledge gaps. There are issues in the treatment of food allergies that need to be addressed, such as education and the quality of life [5,17].

### Aims of the Study

Given the fact that data on food allergy in children in Croatia are not available, the Section for Allergy and Clinical Immunology of the Croatian Pediatric Society initiated this study, as part of the broader investigation on food allergy on the primary level of child care in Croatia. The primary aim of the study was to determine the prevalence of parentally-reported clinical reactions to food and correlation with confirmed allergy. Secondary aims were to determine the parental anxiety about child’s reactions/allergy and the adequacy of managing IgE mediated severe reactions by general pediatricians (GP).

## 2. Patients and Methods

### 2.1. Patients

The study population consisted of children aged 6 to 48 months, sequentially recruited at routine pediatric visits in four pediatric primary care offices in the cities of Zagreb, Bjelovar and Metkovic, from August to October 2014. Primary care for children in Croatia is mandatory, organized in GP offices near the place of living and insurance covers the costs for all children. According to the regulations of Croatian Health Insurance Fund, each GP can have up to 1200 patients 0–7 years old and this was the study base for this research. At the same, visit parents were handed, answered and collected the questionnaire. The only inclusion criterion was child’s age, irrespective of former or actual allergy symptoms.

### 2.2. Methods

#### 2.2.1. Investigators

General pediatricians and a pediatric allergist working in primary care.

#### 2.2.2. The Questionnaire

The questionnaire (Supplement Material) was created by the members of the Section for Allergy and Clinical Immunology (SACI) of Croatian Pediatric Society. The design of the questionnaire is simple, with the questions unequivocally clear and not taking longer than a couple of minutes for parents to answer, in order to have the lowest possible drop out rate. The question about skin test implicates a skin prick test (SPT). Parents whose children had experienced a reaction to food answered the question on their anxiety. The investigation was carried out following the rules of the Declaration of Helsinki of 1975 as revised in 2008. SACI approved the investigation on 30 May 2014. Incomplete or ambiguously answered questionnaires were excluded.

#### 2.2.3. Severe Reactions

Severe reactions (anaphylaxis) were determined based on the consensus clinical definition proposed by Sampson [18] and included (1) the appearance within 2 h of the consumption of culprit food and (2) hives and concomitantly one or more symptoms such as pallor, sudden weakness or floppiness, vomiting, breathing difficulties, rhinorrhea OR sudden weakness and pallor with or without vomiting after consumption of food.

#### 2.2.4. Allergy Workup and Treatment

Allergy workup was done prior to answering the questionnaire and included specific IgE antibodies and/or skin prick test (SPT) and/or open provocation test. In allergy workup children were seen either by general pediatrician only or additionally by a pediatric allergist. General pediatricians could indicate IgE determination, while SPT and provocation tests were carried out at an allergy clinic. Results were interpreted positive if IgE was higher than 0.35 IU/mL and SPT ≥ 3 mm wheal after 15 min, based on EAACI guidelines [19]. Open provocation tests were done in the allergy units or in the pediatric offices. We did not collect data on indications or contraindications for provocation tests. If done in the allergy unit they were based on EAACI guidelines. The indications for allergy workup in the allergy units were not analyzed. Elimination diet, epinephrine autoinjector and education were considered as treatment.

#### 2.2.5. Statistical Methods

Comparison of proportions between groups was performed using the standard chi-square test, or Fisher’s exact test, as appropriate. Differences between groups were compared using *t*-test and analysis of covariance using one or two-way ANOVA. *p* < 0.05 was considered significant. Statistics were calculated using MedCalc Statistical Software [20].

## 3. Results

**Figure 1:**
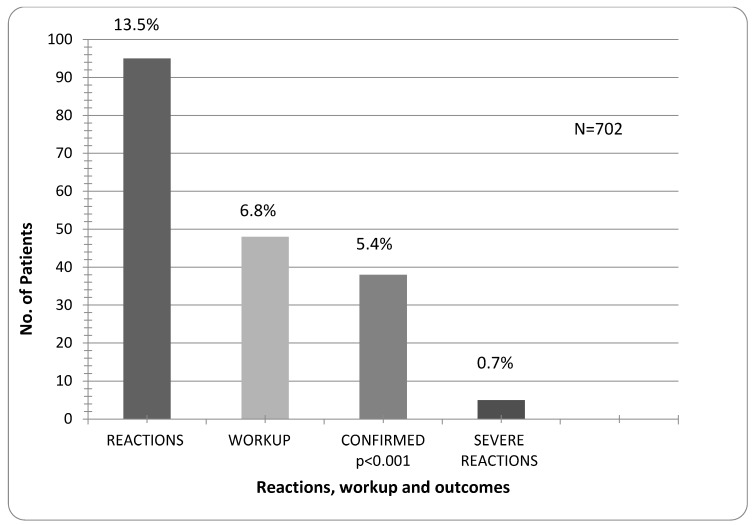
Prevalence of all reactions, allergy workup, confirmed allergy and severe reactions.

The total of 702 properly answered questionnaires was analyzed. Children were grouped in three age groups: 6–12 months (160), 12–24 months (205) and 24–48 months (337). A total of 114 reactions to food were reported in 95 (13.5%) children. Allergy workup was done in 48 (6.8%) and allergy confirmed in 38 (5.4%) children for 60 (52.8%) reactions. 33 patients (4.7%) had hives with or without other symptoms of IgE mediated allergic reaction. In 5 cases (0.7%) the symptoms were apparently primarily non-IgE mediated and appeared within 24 h. Four of five children had deterioration of eczema and positive IgE with/without SPT. One had a bloody stool and positive open provocation test. In 24/38 children workup was done by an allergist—12 children had IgE and SPT, 6 had only SPT, 6 had IgE, SPT and open provocation test. In 14 children workup was done by the general pediatrician—10 had only IgE, one child had IgE and provocation and three children had only provocation. The difference between suspected and confirmed allergy is significant (*p* < 0.001). Severe IgE mediated (anaphylactic) reactions were reported in five of all children (0.7%), or in 13.1% in the allergic group (Figure 1, Table 1).

**Figure 2:**
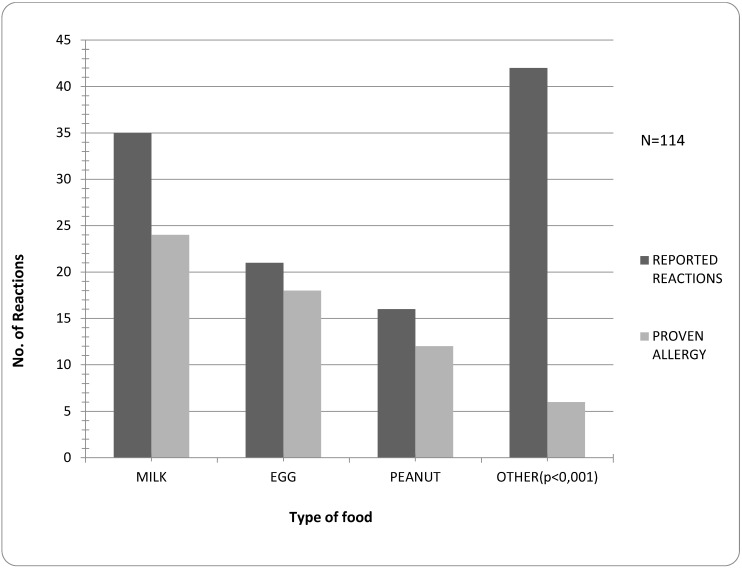
Frequency of reported reactions *versus* proven allergy to types of food.

Fruit and vegetables (apple, pear, carrot, banana, kiwi, cauliflower, ketchup, tomato, cherry, strawberry, citrus fruit), hazelnut, walnut, chicken, soy or “not sure of sort” were most common parentally perceived allergens and, as expected, significantly less confirmed (*p* < 0.001). Besides milk, egg and peanut, allergy was confirmed in single cases for fish, hazelnut, apple and banana. The most common allergen was milk. The difference between suspected and confirmed allergies was not significant for milk, egg and peanut (Figure 2).

**Table 1:** Prevalence of reactions according to the sort of food and age, confirmed allergy and severe reactions.

Age Groups/months	Reactions									Confirmed Allergy		Severe Reactions	
	N	Milk		Egg		Peanut		Other *		N = 702		N = 702	
		N	%	N	%	N	%	N	%	N	%	N	%
6–48	114	35	31	21	18	16	14	42	37	38	5.4		5.2
												5	13.1 **
6–12	28	13	37	5	24	1	6	9	12	9	1.2	1	0.1
12–24	36	9	26	9	43	3	18	15	36	11	1.5	2	0.2
24–48	50	13	37	7	33	12	75	18	43	18	2.5	2	0.2

* Other—apple, pear, carrot, hazelnut, fish, pecan, banana, ketchup, tomato, cherry, chicken, soy, citrus fruit, do not know. ** Severe reactions among children with confirmed allergy.

**Figure 3:**
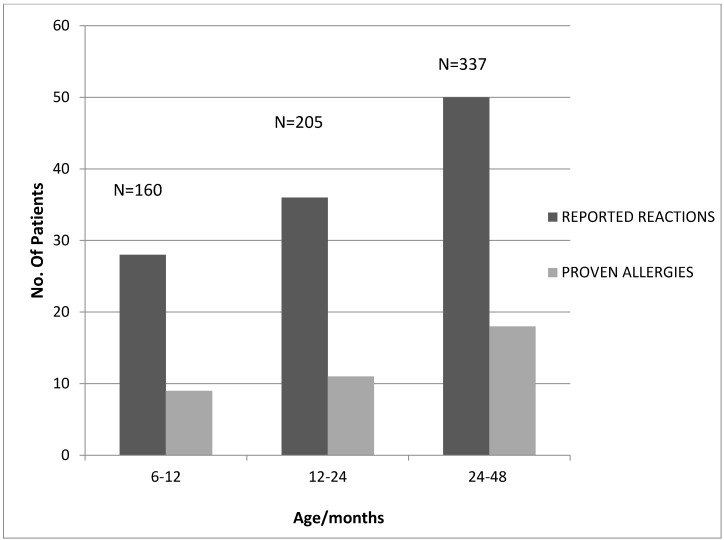
Frequency of reported reactions *versus* proven allergies according to age.

Parental perception of allergic reactions to foods increased with age. However, proven food allergy according to age groups is not significantly different (Figure 3).

**Figure 4:**
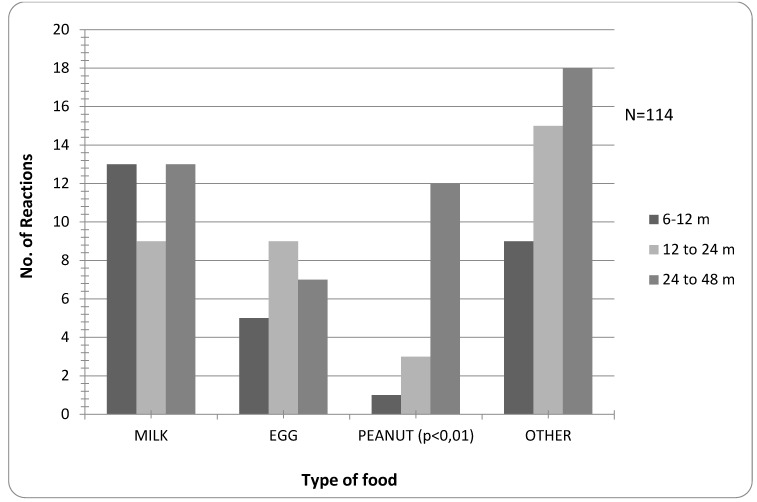
Frequency of suspected reactions to specific food according to age.

There is no significant difference in suspected allergy to certain foods between age groups, except for peanut, which is significantly more frequent in the group ≥2 years (p < 0.01, Figure 4).

**Figure 5:**
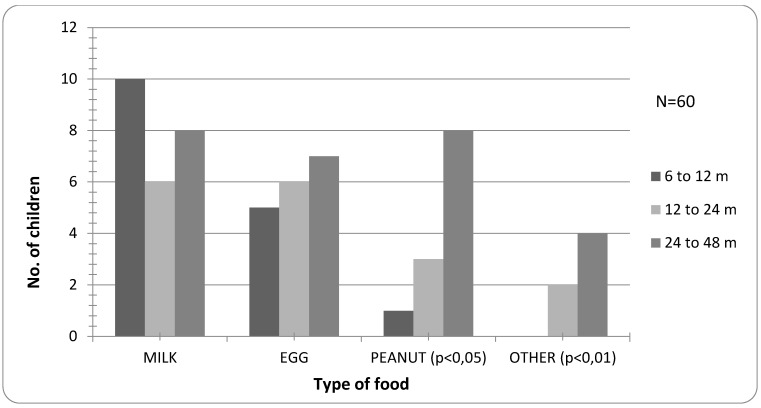
Prevalence of confirmed allergy according to the type of food and age.

Confirmed allergy to peanut is significantly higher in the group ≥2 years (*p* < 0.01), while allergy to foods other than milk, egg or peanut is not present in the group 6–12 months of age (*p* < 0.01) (Figure 5).

Peanut was the culprit food in three out of five patients with severe reactions. One child aged between 12 and 24 months, polisensitized, the other two were older than two years, one polisensitized, and the other monosensitized to peanut. One child had prescribed adrenaline autoinjector. Milk was the cause for severe reaction in an infant and hazelnut in a child between 12 and 24 months. They had been prescribed adrenaline.

All children with clinical reactions had parent-prescribed elimination diet irrespective of proven allergy. Some parents did not even mention the reaction to the child’s physician. Parents of children with clinical reactions to food were *not anxious* about it *at all* in 46/95 (48%) of cases, 45/95 (47%) were *not anxious due to preventive measures*, and 4/95 (4%) were *very anxious*. In the latter group no children had allergy workup done or the allergy was not proven.

Parents of five children with the most severe reactions were *not anxious at all* or *not anxious due to preventive measures taken*.

## 4. Discussion

These are the first results of the prevalence of food allergy based on the population study in Croatia. The reports of prevalence of parentally-reported food allergy vary widely, between 3% and 35% [21] and proven food allergy from 3% to 10% in children younger than four years [6,7,8]. In our study group the prevalence of parentally reported allergy (13.5%) and proven allergy (5.4%) are similar with the observed prevalence in Europe in the same age group [6,10]. The data we collected were not precisely defined and the provocation test, the gold standard for food allergy, was done in the minority of cases. Some parents did not mention the reaction to child’s physician and there was no consistent indication for allergy workup, while some cases negative for IgE/STP were not followed by a provocation test. Due to those limitations, some true allergies may have been missed and the results should be interpreted cautiously. Nevertheless, in the real-life circumstances, we believe that the results for IgE mediated allergy reflect real prevalence. The most common allergen is milk, as recognized by other studies [10,22] Having in mind the overall perception of allergic disease, especially allergy to food, increasing parental perception of allergic reactions to different foods with increasing age is not surprising. The perception is, however, significantly overestimated compared to proven allergy (*p* < 0.001) as has been noted previously [11], particularly to foods other than milk, egg and peanut. Parents’ fear is directed towards a broad range of various foods, even if unspecified. Allergy to peanut is significantly higher in children between 2 and 4 years than in younger children, while allergy to milk and egg in our sample did not differ between these two groups. Milk and egg allergies are known to be most common in the youngest age [10] and in recent work by Dyer [23], who surveyed more than 3000 children with food allergies, the age range with the highest incidence of peanut allergy was 6–10 years. Obviously, a few years are necessary for peanut allergy to develop in most children. Some authors have highlighted the fact that food allergy anaphylaxis is highest in the youngest age group, 0–4 years, which was also the group we investigated. According to medical records, they found anaphylactic reaction to occur in 7 of 100 allergic children [24]. Our results show an even higher prevalence of anaphylaxis, *i.e.*, 13.1% in the allergic children. Peanut was the culprit food in 3/5 of cases.

Parents’ perception of food allergy is clearly overestimating true allergy, as had been shown by other authors [11]. However, once the allergy is confirmed, parents’ anxiety and fear are surprisingly absent. The most common answer to our question on the anxiety about child’s reactions to food was that it was *not* *at all present*, or that it was *absent due to preventive measures*. Only 4 (4%) parents have answered that they *had high degree of anxiety*. Those four parents had children with minor reactions and allergy not proven by the workup. This brings up the problem of parents’ lack of information and education, the major factors for successful dealing with food allergy [5]. Schools for asthma and atopic dermatitis already exist in Croatia, but apparently, a similar patient education project for food allergy would be needed as well. Although our questionnaire was not validated for estimating the quality of life of parents of food allergic children, our results indicate that the anxiety about food allergy is absent in most cases, which suggests an undisturbed quality of life and raises the question of parental understanding of the significance of a child’s condition. Interestingly, as recent work has shown, neither anaphylaxis, nor prescribing an epinephrine autoinjector influences the quality of life in adults or children with food allergy [25]. In optimal circumstances, where there is epinephrine prescribed, there is the problem of parents’ and caregivers’ anxiety of applying it, as recognized in recent work [26]. In our opinion, it points out the need for proper education of parents and caregivers.

In primary care there is a need to address the problem of food allergy due to its frequency. Basic workup should be done on the primary level, without consultation of an allergy specialist, according to the recommendations of treating food allergy in the primary care [27,28]. Our results brought up several problems when dealing with food allergy in primary care—when to indicate allergy workup, what criteria for the exclusion of possible allergy on the basis of clinical history to apply, does elevated IgE after clinical symptoms suffice to start elimination diet and when the workup by an allergist should be done. The fact that some provocation tests were performed in the office without prior workup or allergist consultation, should also pose the question of how to approach to the problem of food allergy. An epinephrine autoinjector was not prescribed in two of five children with severe reactions, which signals the need to improve the competencies of general pediatricians in the field of food allergy. Our pediatricians have periodic trainings in different fields of pediatrics, including on allergies, but we did not collect data on specific training on allergies, which is the limitation for evaluating their competency. Although aware of limitations of the study, some results have so far been obtained. Further studies and more data on food allergy in Croatia are needed.

## 5. Conclusions

Prevalence of food allergy in Croatia appears to be comparable to other European countries. Parents overestimate allergic reactions to food in their children, but when allergy is proven, they tend to underestimate its significance. Preventive measures should include elimination diet and education, as well as an epinephrine prescription with handling where needed. There is a need to work on educating parents and caregivers, but also general pediatricians on food allergies.

## Supplementary Files

Supplementary File 1
