# Supplementary material for: Parental Perception, Prevalence and Primary Care Physicians’ Knowledge on Childhood Food Allergy in Croatia
Source: Children (Basel). 2015 Jul 17;2(3):305–16. doi: 10.3390/children2030305 (PMC4928769; doi:10.3390/children2030305)
Supplement: Supplementary File 1 [file children-02-00305-s001.docx]

The corrections in final proofreading (highlighted in yellow)

1. Dom zdravlja Zagreb-Centar

2. University of Zagreb School of Medicine

3. 38 (5,4%) children (abstract)

4. self-reported (introduction line 8)

5. or floppiness (Methods 2.2.3)

6. pediatricians (Discussion, 4. line from below)
